# Characterization of *Treponema pallidum* Dissemination in C57BL/6 Mice

**DOI:** 10.3389/fimmu.2020.577129

**Published:** 2021-01-08

**Authors:** Simin Lu, Kang Zheng, Jianye Wang, Man Xu, Yafeng Xie, Shuai Yuan, Chuan Wang, Yimou Wu

**Affiliations:** ^1^ Hunan Province Cooperative Innovation Center for Molecular Target New Drug Study, Hengyang Medical College, Institution of Pathogenic Biology, University of South China, Hengyang, China; ^2^ Clinical Laboratory, The Second Affiliated Hospital of University of South China, Hengyang, China

**Keywords:** *Treponema pallidum*, C57BL/6 mice, bacterial dissemination, inflammation, quantitative polymerase chain reaction

## Abstract

The spirochetal pathogen *Treponema pallidum* causes 5 million new cases of venereal syphilis worldwide each year. One major obstacle to syphilis prevention and treatment is the lack of suitable experimental animal models to study its pathogenesis. Accordingly, in this study, we further evaluated the responses of mice to *Treponema pallidum*. Quantitative polymerase chain reaction showed that *Treponema pallidum* could colonize the heart, liver, spleen, kidneys, and testicles of C57BL/6 mice, and the organism may be able to rapidly penetrate the blood-brain barrier in mice by 24 h after infection. In subsequent rabbit infectivity tests, we observed evident signs of the microorganism in the mouse lymph node suspension. After infection, bacterial loads were higher in the tissues than in the blood of C57BL/6 mice. Moreover, a significant Th1 immune response was recorded by cytokine assays. Flow cytometric analysis suggested an obvious increase in the proportion of CD3^+^ T and CD4^+^ T cells in the spleen cells in the infected mice. Thus, improving our understanding of the response of C57BL/6 mice for *Treponema pallidum* will help to comprehensive elucidate the pathogenic mechanisms of this bacterium and lay the foundation for the development of a new research model of *Treponema pallidum.*

## Introduction

Syphilis, a chronic, multisystemic sexually transmitted disease, is caused by the spirochetal bacterium *Treponema pallidum* subsp*. pallidum* (*T. pallidum*). More than 5 million new cases occur annually worldwide, and outbreaks of syphilis often occur in low- and middle-income countries, making syphilis the primary cause of adverse pregnancy and accelerated transmission of acquired immunodeficiency syndrome in these regions (1, 2).

Rabbits are the most commonly used animal model in studies of syphilis because the pathological changes and serological responses of rabbits after infection with *T. pallidum* are similar to those in humans. However, although the bacterium was identified microscopically early in the 20th century, our understanding of the pathogenicity of *T. pallidum* is still limited owing to difficulties in genetic manipulation of rabbits. Additionally, appropriate immune reagents have not been established, making such models even more difficult to establish (3–6).

In contrast, mice, which have a well-defined genetic and immunological background, are frequently used for studies of many infectious diseases. Indeed, many studies of *T. pallidum* infection in inbred mice have been reported. *T. pallidum* has been shown to be able to infect mice and persist within mice. However, infection in mice was not accompanied by skin lesions, as observed in other animal models (7–9), and no further studies have evaluated the merits and demerits of mice as subjects of *T. pallidum*.

The relationship between bacterial dissemination and host immune responses defines the severity of the disease and the outcome of infection. Thus, to further clarify the relationship between *T. pallidum* and different hosts and improve our knowledge of the biology of this organism, we explored the size and kinetics of treponemal burdens in the blood and tissues of mice and the relationship of this organism with tissue pathological injury from the start of the infection.

## Materials and Methods

### Animals

**Figure 1:**
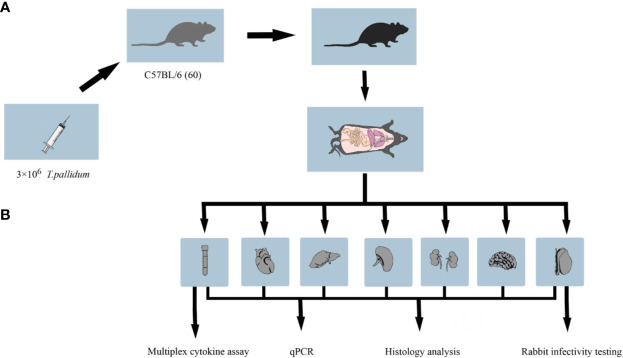
Workflow of the animal experiment. **(A)** C57BL/6 mice in the treatment group were inoculated with 3 × 10^6^ *T. pallidum*. Mice in the control group were injected with 300 μl phosphate-buffered saline. **(B)** Five to six C57BL/6 mice infected with *T. pallidum* were randomly sacrificed at various time intervals after infection, and their blood, heart, liver, spleen, kidney, testis, and brain tissues were collected for DNA extraction, pathological sectioning, and multiplex cytokine assays.

Specific-pathogen-free male mice C57BL/6 (5 weeks old) were obtained from SJA Laboratory Animal Co. Ltd. (Hunan, China). New Zealand white rabbits were a gift from Professor Zhao (University of South China). Antibiotic-free food was provided to mice, and all mice were observed daily for lesions and overall physical appearance. Live *T. pallidum* (Nichols strain) was used for C57BL/6 mice challenge on the same day of extraction from New Zealand rabbit testicles (10). The overall experimental design flow is shown in **Figure 1**.

### Experimental Infection

After acclimation, mice were inoculated with 300 μl virulent *T. pallidum* subsp. *pallidum* (Nichols strain) suspension (3 × 10^6^ total organisms). The inoculation was done by intradermal, intrarectal and corpus cavernosum three sites, each of which was injected with 100 μl virulent *T. pallidum* subsp. *pallidum* (Nichols strain) suspension (11). Equal PBS or equal amounts of testicular extract from uninfected rabbits were injected into the control mice. Five to six mice infected with *T. pallidum* were randomly euthanized at various times after infection (1, 3, 7, 11, 21, 41, 62, 90, 120, and 151 days), and different types of biological samples were collected. To perform rabbit infectivity tests, the inguinal, brachial, and axillary lymph nodes were obtained from mice 11 days post infection for inoculation of New Zealand rabbits, as described previously (11, 12). Based on daily observation of the rabbits and the results of weekly seroconversion tests, rabbits were euthanized at week 9 after inoculation, and testicular tissues were extracted for dark field microscopy detection.

### Extraction of DNA

DNA from blood and tissue samples was extracted using a QIAamp DNA Mini Kit (Qiagen, Shanghai, China) according to the manufacturer’s instructions. DNA from samples was stored at -20°C until analysis by quantitative polymerase chain reaction (qPCR).

### qPCR

qPCR was performed on DNA extracted from blood and tissues from *T. pallidum*-challenged mice according to the manufacturer’s instructions. Primers used for *T. pallidum flaA* (endoflagellar sheath protein, GenBank number M63142) and mouse β-actin were as previously described (13, 14). Quantitative PCR was performed in a 20-μl reaction volumes containing 2 μl DNA, 10 μl of 2× mix, 0.03 μM forward and reverse primers, and 6.8 μl ddH_2_O according to the instructions of the SYBR green I reagent kit (Qiagen, Shanghai, China). All assays were run on a LightCycle 96 apparatus (Roche, Basel, Switzerland). The qPCR conditions for amplification of *flaA* and β-actin were as follows: pre-incubation at 95°C for 10 min; followed by amplification for 40 cycles at 95°C for 15 s, 55°C for 20 s, and 72°C for 20 s; and melting curve analysis for one cycle at 95°C for 10 s, 65°C for 60 s, and 97°C for 1 s. Data analysis was carried out according to the standard curve method. Briefly, a 10-fold serial dilution from 10^7^ to 10^1^ copies of linearized plasmid DNA and a two-fold serial dilution of mouse gDNA from 150 to 1.17 ng/μl were used to construct standard curves for *flaA* and β-actin, respectively (15).

### Pathology

Tissues were fixed in formalin and sent to the University of South China for staining with hematoxylin and eosin. The inflammation was determined by two pathologists, who completed the histopathological evaluation in a blinded manner.

### Multiplex Cytokine Assay

Systemic cytokine responses in mice infected with *T. pallidum*, including serum levels of interleukin (IL)-2, IL-6, IL-10, tumor necrosis factor (TNF)-α, and interferon (IFN)-γ, were evaluated on days 1, 11, and 151 and analyzed using a cytometric bead array (mouse Th1 Panel [5-Plex] kit; BD Biosciences, USA) according to the manufacturer’s protocol. Data were acquired on a BD FACS Canto II flow cytometer (Becton Dickinson, USA) and analyzed using FCAP Array software (Becton Dickinson, USA).

### Immunophenotyping of T Cell Subsets

Spleens were collected from infected mice at day 11 and cell suspension containing 1 × 10^6^ splenocytes were prepared for flow analysis. The cells were stained in accordance with the manual. In short, the cells were incubated with Fc receptor blocking antibodies for 15 min at 4°C after washing twice with FACS buffer containing 1% bovine serum albumin, and stained with surface markers CD3, CD4, CD8, CD62L, and CD44 (eBioscience Inc., CA, USA) in the dark for 30 min at 4°C. After washing twice, the immunophenotype of the T cells were identified by BD FACS Canto II flow cytometer (Becton Dickinson, USA) and the experimental data were analyzed by FACS Diva software (BD Biosciences, USA).

### Statistical Analysis

Two-tailed Student’s t tests were used to compare the *T. pallidum* load (*flaA* DNA copies/μg of mouse DNA) in different organs at the same infection time. GraphPad Prism 7.0 software (San Diego, CA, USA) was used for statistical analysis of the data. Results with *P* values of less than 0.05 were considered statistically significant.

## Results

### Clinical Manifestations of Infection

All C57BL/6 mice tolerated the infection well, without any obvious clinical signs. In contrast, rabbits displayed obvious symptoms of orchitis after 9 weeks injection of the lymph node suspension from infected mice. The specific experimental results from rabbit infectivity tests are shown in **Table 1**.

**Table 1:** Rabbit infectivity testing.

Rabbit testicles inoculated with lymph nodes from mice^a^	Seroconversiona	Darkfield analysis
Control (n = 5)	–	0
Experimental group (n = 5)	+ (Day 20)^b^	5

a The inguinal, brachial, and axillary lymph nodes from mice infected on day 11 were obtained for inoculation of New Zealand rabbits.

b The “+” indicates positive seroconversion [reactive RPR (1:1) and TPPA].

### *T. pallidum* Dissemination in Blood

**Figure 2:**
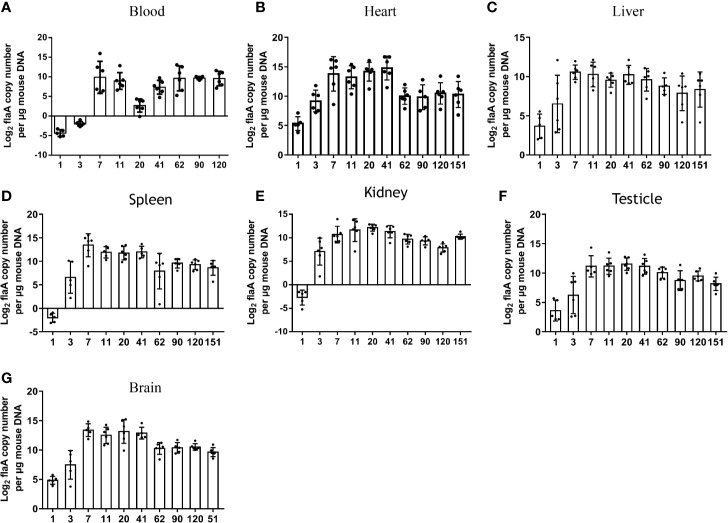
Sizes and kinetics of treponemal burdens in blood and other organs. The *T. pallidum* burden, expressed as log_2_ *flaA* copy number per μg mouse DNA, was evaluated in experimental animals (n = 5 or 6) using qPCR **(A)** Blood, **(B)** Heart, **(C)** Liver, **(D)** Spleen, **(E)** Kidney, **(F)** Testicle, **(G)** Brain. *T. pallidum* was not detected in mice injected with an equal amount of PBS and an uninfected rabbit testicular suspension.

*Treponemal* DNA concentrations (log_2_ *flaA* copies/μg mouse DNA) in blood samples were measured by qPCR at different times after injection. Similar to studies in rabbits, as early as 24 h after injection, *T. pallidum* DNA was detected in the blood of mice (13). As shown in **Figure 2A**, elevated levels of *T. pallidum* DNA were evident on day 3 and reached peak levels on day 7. The increase in bacterial load in infected mice was observed later. As the infection time increase, the microorganism load in the blood was maintained at a relatively stable level. No spirochetes were detected in uninfected group (mice injected with PBS) or in the negative control group (mice injected with an uninfected rabbit testicular suspension) and the amplification curves were shown in **Figure S1**.

### *T. pallidum* Dissemination in Different Tissues

To further explore the burden and dissemination of *T. pallidum* in different tissues during infection, six organs were monitored by qPCR at each time point (**Figures 2B–G**). Despite the fact that a low concentration of bacterial DNA amplification was detected in the six organs at 24 h after inoculation, the *flaA* concentrations in the heart (*P* = 0.0425), liver (*P* = 0.0438), spleen (*P* = 0.020), kidney (*P* = 0.045), and brain (*P* = 0.001) were significantly higher than those in the blood at the corresponding times. The peak in *T. pallidum flaA* amplification in each tissue occurred on day 7 or day 11 after infection. Interestingly, we found that the kinetics of the appearance of spirochetes in all tested tissues was similar, and the burden of bacteria in each organ slowly decreased at different rates, eventually reaching a relatively stable level. However, this trend was not consistent with observations in the blood.

The burdens in various tissues were compared at the same time. Differences in prevalence were observed in different organs. As shown in **Figure 2B**, the mean concentrations of spirochete DNA detected in the heart was greater than that in other tissues at the corresponding time, with the exception of that on day 62. In contrast, the average load of *T. pallidum* in the liver from day 7 after infection was lower than that in other tissues.

### Pathology of the Target Tissue

**Figure 3:**
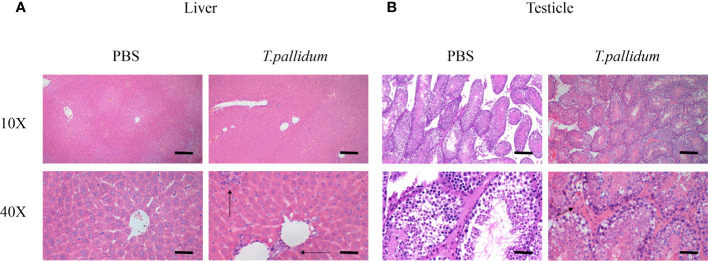
Tissues pathology in mice injected with *T. pallidum*. **(A)** The histopathological changes in the liver of mice 11 days after being attacked by *T. pallidum* were significant. The arrows indicate marked inflammatory cell infiltration in liver tissue. **(B)** The testicles tissue of mice showed relatively mild pathological changes with scattered inflammatory cells on day 11 after injection.

Inflammation was present primarily in the livers of infected mice, as shown in **Figure 3A**. Significant inflammatory changes were not observed in other tissues, including the testicles (**Figure 3B**). Overall, the changes in the infected mice were minimal compared with the histopathology commonly observed in patients with syphilis and in experimentally infected rabbits (data not shown).

### Mild Cytokine Responses Induced by Infection With *T. pallidum*

**Figure 4:**
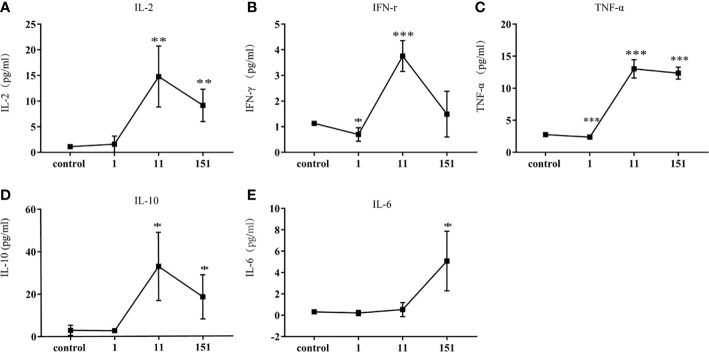
Cytokine responses in C57BL/6 mice following infection with *T. pallidum*. Various cytokines were detected in the serum of C57BL/6 mice on days 1, 11, and 151 after infection. [**(A)** IL-2, **(B)** IFN-γ, **(C)** TNF-α, **(D)** IL-10, **(E)** IL-6]. Data from two independent experiments are shown (n = 3–6, mean ± SD; *P < 0.05, **P < 0.01, ***P < 0.001).

To further characterize the systemic immune responses of mice to *T. pallidum*, we evaluated inflammatory factors in the serum of mice after different times. Th1-related inflammatory cytokines (IL-2, TNF-α, IFN-γ), which are involved in early defense against *T. pallidum*, were detectable in serum within 24 h after infection and reached peak concentrations on day 11. Additionally, IL-10 (a Th2-related cytokine) was significantly upregulated on day 11, which inhibited Th1-related immune responses. In contrast, IL-6 showed only minor upregulation (**Figure 4**).

### *T. pallidum* Infection Induces Proliferation of CD3^+^T Cells and CD4^+^ T Cells in Spleen

**Figure 5:**
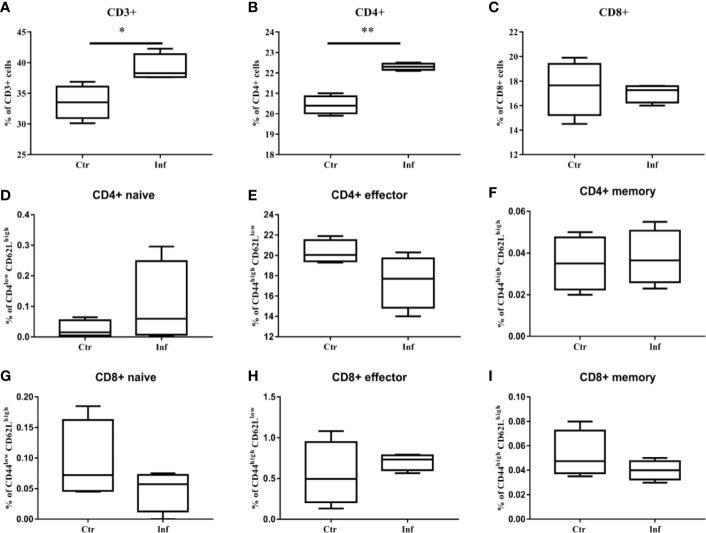
The proliferation of CD3^+^ T cells and CD4^+^ T cells in mice spleen were induced by *T. pallidum*. T-cell subpopulation immunotyping and activation status determination of mouse splenocytes after 11 days of infection were performed by flow cytometry. **(A)** CD3+, **(B)** CD4+, **(C)** CD8+, **(D)** CD4+ naive, **(E)** CD4+ effector, **(F)** CD4+ memory, **(G)** CD8+ naive, **(H)** CD8+ effector, **(I)** CD8+ memory. (Ctr, uninfected mice; Inf, infected mice; n = 4-5; mean ± SD; *P < 0.05; **P < 0.01).

The proliferation of specific splenocytes in mice was detected after 11 days of *T. pallidum* infection. Compared with the control group, Flow cytometric analysis showed that that the proportion of CD8^+^ T cells decreased (**Figure 5C**) when there was a significant increase in the proportion of CD3^+^ T cells and CD4^+^ T cells (**Figures 5A–B**) in the spleen cells in the infected group. In addition, we examined the expression of CD44 and CD62L in CD4+ T cells and CD8+ T cell populations because CD44 was upregulated during effector T cells activation, whereas CD62L was downregulated. It is puzzling that, in our experiments, no significant changes in the proportion of CD44^high^ CD62L^low^ in CD4^+^ T cells and CD8^+^ T cells were observed (**Figures 5D–I**).

## Discussion

*T. pallidum* causes systemic infection in humans and experimental rabbits. Different animal models have been tested, including mice; however, it seems that no model has been shown to be better than rabbits. Moreover, except for two studies (7, 16), no reports have demonstrated the development of cutaneous lesions after *T. pallidum* inoculation in mice. Notably, this bacterium can persist in mice and be transferred to rabbits by lymph node inoculation (8). Therefore, here, we studied the spreading trend of *T. pallidum* in mice and triggered immune response. In all challenged animals, no mice showed macroscopic lesions at the inoculation site or any other site, although *T. pallidum* DNA was detected in different tissues by qPCR. Subsequent rabbit infectivity tests also showed signs of microorganisms in lymph node mixtures in infected mice. These phenomena are consistent with previous studies, indicating that we succeeded in infecting C57BL/6 mice.

The reason for the lack of obvious clinical manifestations in mice infected with *T. pallidum* is complex and unclear. The pathogenicity of bacteria and the interaction between the host immune response determine the severity of the disease and the outcome of infection. As described by Folds and coworkers, when mice were infected with *T. pallidum* in which the outer membrane was incorporated with rabbit protein or host lipids, a suitable microenvironment was not established to permit the pathogenicity of the microorganism (7, 8, 16–18). Thus, the pattern observed in mice may be similar to that occurring in humans during latent infection. In view of latency can revert to fulminant infection in humans, as observed in secondary syphilis and some types of tertiary syphilis; we performed tests on mice from day 1 to day 151. During this period, however, the mice maintained a healthy appearance except for changes in the load of *T. pallidum* in the organs.

Within hours to days, *T. pallidum* is able to disseminate throughout various tissues from the infection site (19–22), and our mice in this study showed similar characteristics. Strikingly, the average concentration of *T. pallidum* DNA in mouse tissues was always higher than that in whole blood at the same time point. This feature, which was not often detected in rabbit models, was consistent with the results of studies in other pathogens in mice. Moreover, different inoculation methods affect both the pathogenicity and tropism of pathogens (13, 23–26), and dynamic temporal and spatial regulation of *T. pallidum* genes is important for its successful colonization, dissemination, and invasion in hosts (27). In this study, however, we did not detect *T. pallidum* gene expression levels. We also showed that the contents of *T. pallidum* were higher in the heart and spleen, suggesting that these organs may be more inclined to provide *T. pallidum* with the necessary material for survival (28). Moreover, the lack of heat shock response regulated by σ32 may hamper the attachment and replication of *T. pallidum* in the liver when the internal temperature is too high for optimal growth (29, 30). This phenomenon was consistent with the results of Salazar’s experiments on New Zealand rabbits, but there is a difference with the results of Silver’s experiments on mice (11, 13).

Neurosyphilis, a major complication that may causes death in patients with syphilis, can occur at any time after infection. Studies have shown that *T. pallidum* can break through the blood-brain barrier at the beginning of infection and colonize the brain tissue in both humans and experimental rabbits. Tp92 and Tp0751 may be involved in this process by mediating the adhesion of *T. pallidum* to host cells, although its exact mechanism needs to be further elucidated (31, 32). Our results suggested that the brain may be an important site for spirochetal invasion, demonstrating consistent PCR positivity. But obvious pathological changes were not recorded in mouse brain tissue. Given the method of inoculation used in the study, unfortunately, it is not clear whether spirochetes in the brain are caused by the invasion of the spirochetes themselves or by blood transmission. Changes in the load of *T. pallidum* in the blood and tissues are thought to be the result of competition between the ability of *T. pallidum* to avoid immune recognition and the adeptness of the host’s innate and adaptive immune responses to track down and eliminate the spirochetal pathogen. In the past few decades, cellular immune responses, macrophage activation, and opsonic antibody production have been shown to be effectors of *T. pallidum* clearance (33–35). The concentration of *T. pallidum* in the blood first decreased significantly after infection in our experiment, which may indicate that activation of immune responses in the blood occurred earlier or stronger than that in tissues. Mild changes in treponemal DNA levels in tissue samples were consistent with the observed serological responses in the mice. Although humoral immune responses have not yet been tested, James’s experiments have indicated that the humoral response in mice is slower than that in rabbits to *T. pallidum* (36). Furthermore, previous studies have indicated that the presence of spirochetes within tissues not only constitutes the driving force for the activation of resident immune cells but also recruits immune effector cells from peripheral blood (37). Therefore, the increase observed in the blood could be the result of immune cell recruitment.

Previous studies have confirmed that *T. pallidum* can trigger a strong immune response after infecting the host. In addition to macrophage activation and mononuclear cell infiltration, high levels of Th1 inflammatory factors with bactericidal activity could be detected in the early stage of infection. In our system, an increase in the proportion of CD4+ T cells in spleen cells and the parallel elevation of IL-2, IFN-γ, and TNF-α in serum after 11 days of infection in mice suggested that Th1 immune responses may be caused by early infection by *T. pallidum*. This result may indicate similar significance to Centurion-Lara and Van experiments. The former showed that Th1 inflammatory factors were closely related to the temporary recovery of primary and secondary syphilis patients, while the latter showed that Th1 inflammatory factors were with early recovery of rabbit lesions (38, 39). However, our experimental data were taken together showed the immune response in mice does not seem to clear the host of *T. pallidum*. There was no significant increase in either the CD4+ effector or the CD8+ effector, which may be closely related to the slow decrease in *T. pallidum.* Meanwhile, the increases in IL-10 and IL-6 may be important reasons for the inability of Th1 immune responses to eliminate *T. pallidum* despite significant inhibition of the inflammatory reaction. Overall, the inflammatory response in C57BL/6 mice after infection was mild, which may provide a partial explanation for the absence of gross manifestations in mice after infection with *T. pallidum*.

In summary, we successfully infected C57BL/6 mice with *T. pallidum* and studied the dissemination and pathogenesis of *T. pallidum* in mice. Our study provided insights into the use of mice as a model for studies of syphilis, particularly asymptomatic syphilis with no obvious external lesions. Unfortunately, in this experiment, the dissemination of *T. pallidum* and its cause of inflammation were not compared in rabbits and mice. The comparison results will help us to better understand the relationship between the *T. pallidum* and the host, and thus the pathogenicity of bacteria. PCR was the only technique used to detect *T. pallidum* and more experimental methods need to be adopted to confirm the results.

## Data Availability Statement

The raw data supporting the conclusions of this article will be made available by the authors, without undue reservation.

## Ethics Statement 

The animal study was reviewed and approved by The animal welfare committee of the University of South China.

## Author Contributions

SL, KZ, and MX designed this experiment, and the experiment was completed by SL and Wang. The statistics of the experimental data were done by YX, SY, and Wang together. All authors contributed to the article and approved the submitted version.

## Funding

This work was supported by the National Natural Science Foundation of China (Grant numbers 81702046), the Hunan Province Cooperative Innovation Center for Molecular Target New Drug Study (2015–351), and the General Project in Hunan Province Science and Technology Program (Grant number 2014TT2025).

## Conflict of Interest

The authors declare that the research was conducted in the absence of any commercial or financial relationships that could be construed as a potential conflict of interest.

## Supplementary Material

Supplementary Figure 1 The amplification curves of samples from some uninfected and negative control groups. **(A)** the uninfected group; **(B)** the negative control group.
