# Supplementary figures and images for: Characterization of Treponema pallidum Dissemination in C57BL/6 Mice
Source: Front Immunol. 2021 Jan 8;11:577129. doi: 10.3389/fimmu.2020.577129 (PMC7819853; doi:10.3389/fimmu.2020.577129)

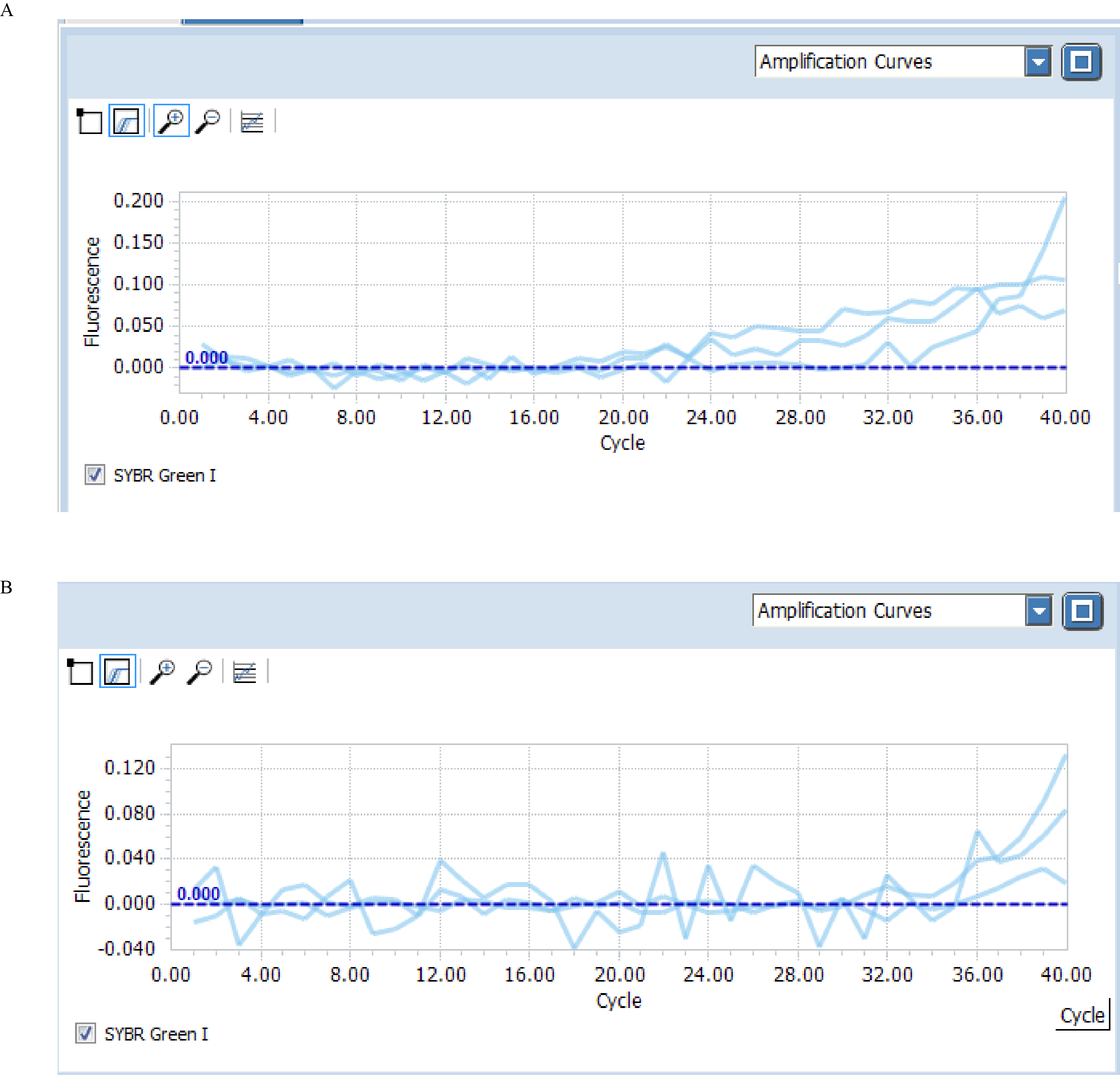

Supplement: Supplementary Figure 1 — The amplification curves of samples from some uninfected and negative control groups. (A) the uninfected group; (B) the negative control group. [file Image_1.tif]
